# Prognostic significance of heat shock protein 70 (HSP70) in patients with oral cancer

**DOI:** 10.1186/1758-3284-3-10

**Published:** 2011-02-23

**Authors:** Frank Tavassol, Oliver F Starke, Horst Kokemüller, Gerd Wegener, Corinna CM Müller-Tavassol, Nils-Claudius Gellrich, André Eckardt

**Affiliations:** 1 Department of Oral and Maxillofacial Surgery, Hannover Medical School, Hanover, Germany; 2 Department of Cranio- and Maxillofacial Surgery, Heinrich-Heine-University, Duesseldorf, Germany; 3 Tumor Registry, Hannover Medical School, Hanover, Germany; 4 Department of Gastroenterology, Hepatology and Endocinology, Hannover Medical School, Hannover, Germany

## Abstract

**Backround:**

Oral squamous cell carcinoma (OSCC) is characterized by an aggressive growth pattern, local invasiveness, and spread to cervical lymph nodes. Overall survival rates have not improved, primarily due to locoregional tumor recurrences and distant metastasis. To date, no trustworthy or clinically applicable marker of tumor aggressiveness has been identified for OSCC. Heat shock proteins (HSPs) play a role in tumor antigenicity. This study aimed to investigate the expression and prognostic significance of highly stress-inducible HSP70 in OSCC.

**Methods:**

Immunohistochemical staining for HSP70 was performed on surgical specimens obtained from 61 patients with OSCC. Light microscopy and analysis 3.1^® ^(Soft Imaging System, Münster, Germany), an image processing and analysis program, were used for evaluating HSP70 expression. The tumor region was defined as the region of interest (ROI) and HSP70-positive staining was analyzed.

**Results:**

Immunoreactivity for HSP70 was positive in tumor cells of 38 of all patients (63.3%). Positive immunoreactivity of tumor cells could be detected in 17 of 28 patients with T2 tumors (60.7%) Prognostic significance of HSP70 expression in tumor cells was detected in patients with T2 tumors (p = 0.009).

**Conclusions:**

The survival of patients suffering from T2 tumors with positive HSP70 expression was 8 times higher than that for patients with negative HSP70 expression, suggesting that T1-T2 tumors of OSCC with low expression of HSP70 require more radical treatment.

## Background

Oral squamous cell carcinoma (OSCC), a frequently occurring cancer in the head and neck region, is characterized by an aggressive growth pattern, local invasiveness, and spread to cervical lymph nodes. Patient outcome depends on the conventional prognostic factors used in clinical practice. Advances in surgical and nonsurgical treatments have led to increased local tumor control in recent years. However, overall survival rates have not improved because of the prevalence of locoregional tumor recurrence and distant metastasis. Although there is general agreement that tumor infiltration of the resection margin is one of the most relevant predictive factors for the development of a local recurrent carcinoma, the presence of tumor-free margins does not guarantee against recurrence because carcinoma can develop following discontinued expansion of tumor cells in the vicinity [1,2]. To improve survival periods of these patients, molecular and histological markers must be identified to target tumors with a high likelihood of metastatic spread. To date, no reliable or clinically applicable marker of tumor aggressiveness has been identified for OSCC. Different markers/marker complexes have been identified as active in tumor suppression or antitumor defense and display potential as prognostic factors. Furthermore, molecular biology investigations of resection margins have shown that detection of mutant p53 genes is linked with increased incidence of recurrent tumors. The p53 molecule is a 53-kD polypeptide. It acts as a transcription factor that controls the cell cycle by either arresting cells in the G1 phase through activation of the p21 gene or triggering apoptosis by activating genes. Another more recent approach to oral carcinogenesis focuses on the escape of malignant cells from apoptotic signals. Extensive research has been carried out on p53 in this respect, and there is broad evidence for its role in the manifestation of oral carcinoma. However, published data indicates that p53 alone is not particularly valuable in predicting prognosis. Additional markers of apoptosis such as Fas, Fas ligand (FasL), and Bax, as well as anti-apoptotic molecules such as bcl2/BAG-1, are reported to be relevant to prognosis in a smaller number of publications. All of these have shown a significant correlation with prognosis, but it is difficult to draw conclusions on the prognostic validity of these markers on the basis of the present data [3-5].

Another approach in predicting prognosis in cancer is expression of heat shock proteins (HSPs). HSPs are found in all organisms and all cell types. They are the most phylogenetically conserved proteins known with respect to both structure and function [6]. Usually, HSPs are expressed at low levels, and under normal physiological conditions, many members of the HSP family are involved in protein synthesis. When a cell is stressed, oligomeric complexes disassemble and polypeptides unfold. Under these conditions, the role of HSPs is to reverse such changes and, if refolding becomes impossible, to potentially speed up the removal of such denatured proteins. Expression of HSPs is induced even under nonstress conditions, including those of the cell cycle, development, and differentiation [7,8]. During carcinogenesis, HSPs have been reported to alter their expression levels, showing either an increase or a decrease [9,10]). HSP70 expression in colorectal carcinoma and breast carcinoma has been significantly correlated with low differentiation and poor prognosis [11,12], whereas in renal cell carcinoma it has been reported to be associated with good prognosis [13]. Five main families of HSPs are known: low molecular weight, HSP65, HSP70, HSP90, and HSP100. Over the last few years, HSPs have also been shown to play a role in the antigenicity of tumors. Expression of HSPs on the surface of tumor cells, instead of their normal intracellular location, suggests they play a role in inducing an immune response against cancer. In a chemically induced mouse sarcoma, Ullrich et al. identified a tumor-specific transplantation antigen that appears to be an HSP, which is expressed on the cell surface and induces protective immunity [14]. Furthermore, a protein related to the HSP70 family has been shown to be immunogenic in oncogene-transformed rat fibroblasts [15], and HSP70 derived from MethA sarcoma (but not from normal tissue) has been demonstrated to be immunogenic, not in itself but in association with tumor peptides. The prognostic significance of HSP70 in esophageal squamous cell carcinoma has been reported [16]. The aim of the present study was to investigate the expression of highly stress-inducible HSP70 in OSCC and its use in predicting prognosis.

## Methods

### Patients

Surgical specimens were obtained from 61 patients (49 men and 12 women) with OSCC who underwent potentially curative surgery at the Department of Oral and Maxillofacial Surgery, Hanover Medical School, Hanover, Germany, between 1996 and 2001. Tumor stage and disease grade were classified according to the fifth edition of the TNM classification of the International Union Against Cancer (UICC). None of the patients had received irradiation or chemotherapy prior to surgery or had distant metastases at the time of surgery. Patients who underwent no curative surgery and/or inadequate follow-up were not included in the study.

### Immunohistochemistry

Serial 3-mm sections were deparaffinized, rehydrated, washed and, treated with a solution of 2% horse serum, 0.1% bovine serum albumin (Sigma Corporation, Steinheim, Germany), and 0.1% sodium acid in 150 mmol/l phosphate-buffered saline (PBS; pH 7.2) for 15 min to block nonspecific antibody-binding. A polyclonal rabbit anti-HSP70 antibody (Dako, Carpinteria, CA, USA), specific to HSP from *Escherichia coli*, which shares more than 48% sequence homology with mammalian HSP70 was the first layer. The optimal dilution of anti-HSP antibody (1:250) was determined by titration. The selected sections were incubated with this antibody for 120 min at room temperature (RT). The second layer, a biotin-conjugated goat anti-rabbit immunoglobulin (Oncogene, San Diego, CA USA) diluted 1:200 in PBS was incubated for 30 min at RT. The third layer was an avidin-biotin-horseradish peroxidase complex (Dako) diluted 1:50 in PBS. Incubation was, as before, 30 min at RT. Sections were washed for 10 min in 2 changes of PBS between each layer. The color reaction was developed with a solution consisting of 0.05% 3,30-diaminobenzidine tetrahydrochloride (Sigma, St Louis, MO, USA), 0.03% nickel chloride (Sigma), and 0.01% hydrogen peroxide in 48 mmol/l Tris-HCL, pH 7.6 (Sigma). Counterstaining was carried out with Mayer's hematoxylin [6].

### Evaluation of HSP70 Expression

Light microscopy and analysis 3.1^® ^(Soft Imaging System, Münster, Germany), an image processing and analysis program, were used for evaluating HSP70 expression. The tumor region was defined as the region of interest (ROI) and HSP70-positive staining was analyzed. When 20% or more of the tumor cells in a given specimen were positively stained, the sample was graded as HSP70 positive; it was graded negative when fewer than 20% of the tumor cells were stained [16].

### Evaluation of Clinicopathological Parameters

Histological evaluation was performed using hematoxylin and eosin staining. TNM-classification was applied with regard to the clinical stage, depth of tumor invasion, lymph node status, and histological typing using the World Health Organization classification.

### Statistical analysis

The statistical significance of the data was analyzed using the chi-square test. Patients' postoperative status was assessed on December 31, 2008. No patient with incomplete follow-up was included in the evaluation. The cumulative survival rate was calculated by the Kaplan-Meier method [17], and statistical significance was analyzed by the log-rank test. To confirm the statistical significance of HSP70 expression as a prognostic indicator, multivariate analysis was performed using the Cox proportional-hazards regression model [18].

## Results

### Clinical and pathological features

Of the 61 patients, 49 were men and 12 women. The age of the patients ranged from 35 to 60 years with a mean age of 50.7 years. T2 tumors were present in 28 patients, T3 tumors in 3 patients, and T4 tumors in 30 patients. Lymph node metastases were found in 25 patients.

### Clinicopathological findings and survival

**Figure 1:**
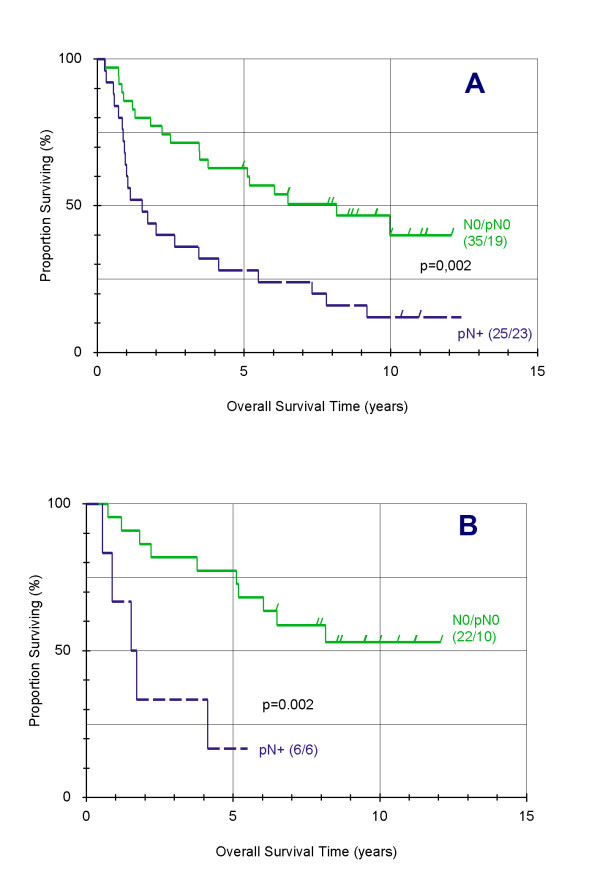
**Influence of nodal stage on overall survival following diagnosis of oral cancer, using the product-limit-method of Kaplan and Meier**. Considering all patients (A), 25 of 35 had a positive lymph node stage. Regarding the patients suffering from T2 tumors (B), 6 of 28 showed a positive lymph node stage. Survival of patients with negative lymph nodes (green) and positive lymph nodes (blue) (p = 0.002).

There was no statistical influence on prognosis of grading, T- stadium, age, and sex of the patients. Presence of positive lymph nodes (N+) was identified as a prognostic factor with significant influence in univariate and multivariate analysis, as well in all patients suffering from T2 tumors (Table 1, Figure 1A, and Figure 1B). The mortality risk of these patients is 13 times than that of patients with negative nodes.

**Table 1:** Expression of HSP70 and other tumor characteristics multivariate analyses for disease-free survival prediction in 28 patients with T2 oral squamous cell carcinoma.

Covariable	Values	**n**_**1**_**/n**_**2**_	p	RR	95%-CI[Iwr,upr]
Sex	m/f	23/5	n. s.	-	

Age	<50/≥ 50	10/18	n. s.	-	

pN	N0/pN0/pN+	22/6	0.009	13.070	[1.912, 89.354]

Grading	G1-G2/G3	23/5	n. s.	-	

HSP70	<20%/≥20%	11/17	0.010	0.123	[0.025, 0.607]

(Cox's partially nonparametric regression model was used to evaluate the predictive power of various combinations and interactions of prognostic factors. Cl = confidence interval.)

### Immunohistochemistry of HSP70

**Figure 2:**
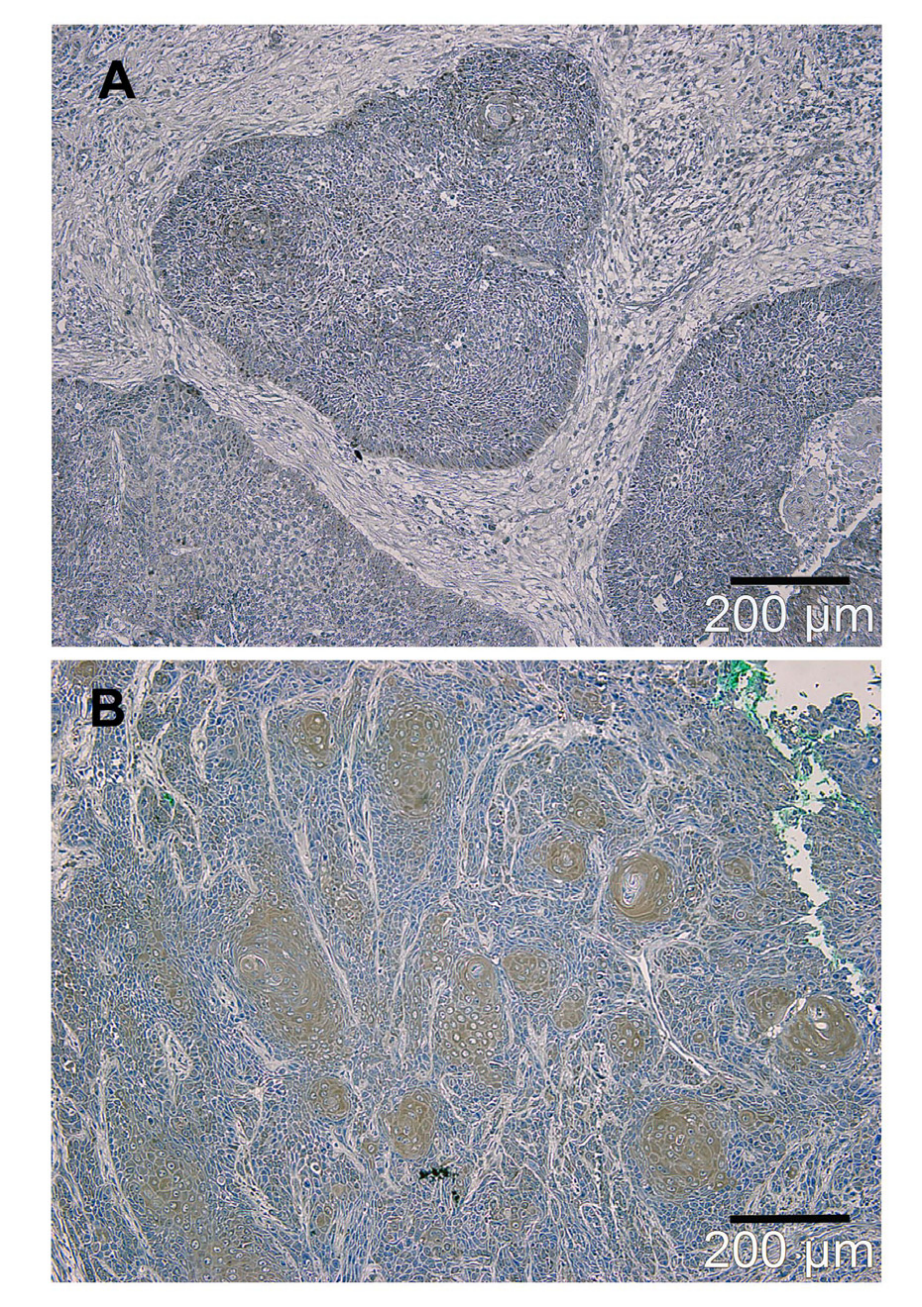
**Photographs from tissue sections of oral squamous cell carcinoma immunostained for HSP70**. Expression pattern of HSP70-negative (A) and HSP70-positive (brown color, B) specimens.

Immunoreactivity for HSP70 was positive in tumor cells of 38 of all patients (63.3%). Positive immunoreactivity of tumor cells could be detected in 17 of 28 patients with T2 tumors (60.7%) (Table 2, Figure 2).

**Table 2:** Proportional distribution of HSP70-positive and HSP70-negative tumors separated into T2 and T3/T4 tumors.

	HSP 70 positive	HSP 70 negative
T2 (n = 28)	17	11

T3/T4 (n = 33)	21	12

### Prognostic significance of HSP expression

**Figure 3:**
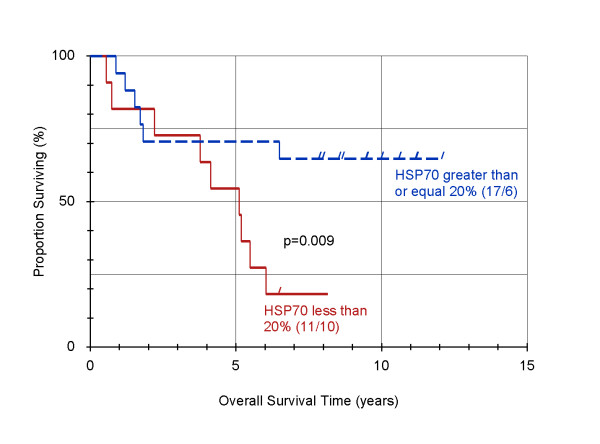
**Influence of HSP70 expression on overall survival of patients suffering from T2 tumors following diagnosis of oral cancer, using the product-limit-method of Kaplan and Meier**. Of 28 patients, 17 patients showed HSP70 expression greater or equal to 20% (blue curve). This predicts significantly improved survival compared to patients with less then 20% expression of HSP70 (red curve). [p = 0.009]

When all patients were considered, no statistical influence on survival of HSP70 could be detected. After classifying the samples as T2 and T3/T4 tumors, prognostic significance of HSP70 expression in tumor cells could be detected in patients suffering from T2 tumors. Figure 3 shows cumulative survival curves for patients with T2 tumors with positive and negative HSP70 expression (Table 1 and Figure 3).

The survival of patients suffering from T2 tumors with positive HSP70 expression was 8 times higher than that for patients with negative HSP70 expression.

## Discussion

Many studies in cell biology have addressed the roles of HSPs as molecular chaperones, including protein folding and unfolding, translocation, and prevention of inappropriate protein aggregation [7,19]. These findings show an association between HSPs and cell proliferation and the prevention of apoptosis [20]. While HSP expression has been recognized as a factor of prognostic value in certain tumors, the data are limited and the results often contradictory. For example, inducible HSP72 has been shown to be a negative prognostic factor for disease-free survival (DFS) in patients with lymph node-negative breast cancer [21], whereas other authors have shown that HSP72 positively correlates with estrogen receptors and inversely with expression of mutant p53 [22]. Santarosa et al. demonstrated prognostic implications of expression of HSP70 in patients suffering from renal cancer [23]. Although some evidence indicates that HSPs are involved in various aspects of cell transformation and immune response against cancer [24], their biological role and its implications for the clinical course in cancer patients are not clear. In OSCC, the number of positive nodes, macroscopic extracapsular spread, and tumor infiltration of the resection margins have been described as significant prognostic factors [1,2]. Studies concerning HSP70 as a prognostic factor in esophageal carcinoma suggest that reduction of HSP70 expression is significantly correlated with poor prognosis [16,25]. Although several studies have been performed to elucidate the relationship between HSPs and tumors in various organs, to our knowledge, there are comparatively few reports related to OSCC. Sugarman et al. reported that HSP70 expression is not a definitive marker of oral malignancy or malignant potential [26]. In terms of prognostic significance, Ito et al. examined 24 specimens of patients suffering from OSCC. Although HSP immunohistochemistry revealed changes in HSP expression during tumorigenesis of squamous epithelium of the tongue, there was no correlation between HSP staining and survival period, stage, lymph node metastasis, histological grade, or p53 immunostaining [27]. These results are in line with those of Gandour-Edwards et al, who found that HSPs were expressed in normal upper respiratory tract squamous mucosa, and their role in carcinoma thus remains unclear. None of the markers (p53, HSP27, or HSP70) demonstrated prognostic significance for 5-year survival. We confirm the previously identified association of cervical lymph node metastases with decreased survival [5].

The results of the previously published data are not in line with the findings of the present study. In this study, we demonstrated that for patients suffering from T2-tumors, positive expression of HSP70 results in a significantly lower mortality risk compared with patients with negative expression. The main difference regarding the study design may be that, in contrast to previous studies, our patient population was divided according to tumor stages in the different T-stadium of the tumor. The purpose of this division was to analyze the prognostic significance of each T-stadium separately. Considering the entire patient group, our findings would suggest the same result: HSP70 has no prognostic implications for our patients. Also in the seperated T3- and T4-tumors, HSP 70 did not show any prognostic significance. Only after focusing on patients suffering from T2-tumors could a significant difference be detected. The survival of patients suffering from T2 tumors with positive HSP70 expression was 8 times higher than that for patients with negative HSP70 expression.

## Conclusions

The results of our study suggests that expression of HSP70 affects survival only in the early stage of the disease and that HSP70 membrane expression, in particular, is a target for natural killer cells [28]. Therefore in T2-tumors, the increased level of HSP70 may result in an extended tumor control by the natural killer cells. By implication, T1-T2 tumors of OSCC with low expression of Hsp70 could require more radical treatment.

## Competing interests

The authors declare that they have no competing interests.

## Authors' contributions

FT, HK, NCG and AE conceived of the study and participated in its design and coordination. FT drafted the manuscript. CMT helped to draft the manuscript. OFS carried out the immunohistochemistry and the HSP 70 Expression. GW performed the statistical analysis. All authors read and approved the final manuscript.
